# Functional D-box sequences reset the circadian clock and drive mRNA rhythms

**DOI:** 10.1038/s42003-019-0522-3

**Published:** 2019-08-08

**Authors:** Hikari Yoshitane, Yoshimasa Asano, Aya Sagami, Seinosuke Sakai, Yutaka Suzuki, Hitoshi Okamura, Wataru Iwasaki, Haruka Ozaki, Yoshitaka Fukada

**Affiliations:** 1 Department of Biological Sciences, School of Science, The University of Tokyo, Hongo 7-3-1, Bunkyo-ku Tokyo, 113-0033 Japan; 2 Department of Medical Genome Sciences, Graduate School of Frontier Sciences, The University of Tokyo, Kashiwanoha 5-1-5,, Kashiwa Chiba, 277-8568 Japan; 3 Department of Systems Biology, Graduate School of Pharmaceutical Sciences, Kyoto University, Yoshida-Shimo-Adachi-cho 46-29, Kyoto, 606-8501 Japan; 4 Bioinformatics Laboratory, Faculty of Medicine, University of Tsukuba, Tennodai 1-1-1, Tsukuba, Ibaraki, 305-8575 Japan; 5 Center for Artificial Intelligence Research, University of Tsukuba, Tennodai 1-1-1, Tsukuba, Ibaraki, 305-8577 Japan

**Keywords:** Gene regulation, Circadian rhythms, Circadian mechanisms

## Abstract

The circadian clock drives gene expression rhythms, leading to daily changes in physiology and behavior. In mammals, Albumin D-site-Binding Protein (DBP) rhythmically activates transcription of various genes through a DNA cis-element, D-box. The DBP-dependent transactivation is repressed by competitive binding of E4BP4 to the D-box. Despite the elaborate regulation, physiological roles of the D-box in the circadian clockwork are still elusive. Here we identified 1490 genomic regions recognized commonly by DBP and E4BP4 in the mouse liver. We comprehensively defined functional D-box sequences using an improved bioinformatics method, MOCCS2. In RNA-Seq analysis of *E4bp4*-knockout and wild type liver, we showed the importance of E4BP4-mediated circadian repression in gene expression rhythms. In addition to the circadian control, we found that environmental stimuli caused acute induction of E4BP4 protein, evoking phase-dependent phase shifts of cellular circadian rhythms and resetting the clock. Collectively, D-box-mediated transcriptional regulation plays pivotal roles in input and output in the circadian clock system.

## Abstract (web summary)

Hikari Yoshitane, Yoshimasa Asano et al. investigate role of the D-box genomic element in the circadian clockwork and identify 1490 D-box-containing regions that are bound by DBP and E4BP4 in the mouse liver. Using mice lacking liver expression of *E4bp4*, they demonstrate the importance of the D-box element for mediating environmental input response and resetting the circadian clock.

## Introduction

Many aspects of animal behavior and physiology show regular patterns based on circadian rhythms, and these rhythms are observed in a wide range of organisms^1^. Circadian rhythms are governed by the circadian clock system, which is composed of three components: an oscillator that oscillates even under constant conditions; an input that allows the oscillator to synchronize with environmental cycles; and an output that transmits the oscillator’s signals into circadian gene expression and physiological rhythms. In the circadian oscillator, clock genes and their encoded proteins form transcriptional/translational feedback loops, and drive expression rhythms of core clock genes^2^. In mammals, CLOCK and BMAL1 bind to a DNA cis-element E-box to transactivate a wide range of target genes including their negative regulators, *Per* and *Cry* genes. In addition to the E-box element, D-box element and REV-ERB/ROR-binding element (RRE) form a regulatory network of the rhythmic gene expression, governing coordinately the transcriptional oscillations^3,4^. The D-box element is activated by three members of the PAR bZip family: Albumin D-site-Binding Protein (DBP); Thyrotroph Embryonic Factor (TEF); and Hepatic Leukemia Factor (HLF). D-box-dependent transactivation is repressed by a bZip factor, Adenovirus E4 promoter Binding Protein 4 (E4BP4), also referred to as Nuclear Factor Interleukin 3 regulated (NFIL3)^3,5^.

DBP was originally identified as a transcription factor that binds to the D-site in the promoter region of the *Albumin* gene^6^. DBP activates transcription of the *Per1* gene by binding to the promoter region, and mutation of a putative DBP-binding site abolishes the DBP-dependent transactivation^7^. E4BP4 protein represses the DBP-dependent transactivation by its competitive binding to the same DNA sequence^5^. A pioneering work in the field of circadian system biology defined TTAYGTAA as the D-box motif, and showed rhythmic expression from reporter constructs including the D-box sequences^3^. Furthermore, the circadian peak phase of the D-box activity is located between those of the E-box and RRE activities, and combinations of the three DNA cis-elements in the gene loci determine gene expression profiles^3,8^. Thus, D-box-mediated transcriptional regulation appears to be important for the circadian clockwork, but physiological roles of D-box sequences in the clock system are still elusive. Moreover, the D-box motif has been extracted from a limited number of genes, and hence a comprehensive analysis is required to determine functional sequences that serve as the D-box in vivo. Here, chromatin immunoprecipitation (ChIP)-Seq analysis in mouse liver and an improved bioinformatics method termed MOCCS2 defined functional D-box sequences, among which TTATGCAA and TTATGTAA are the most and second-most preferred sequences, respectively. Furthermore, we found that acute induction of E4BP4 protein caused phase resetting of peripheral clocks, indicating the importance of D-box function not only in the output but also in the input of the circadian clock system.

## Results

### Genome-wide analysis of DBP-binding and E4BP4-binding sites

**Fig. 1:**
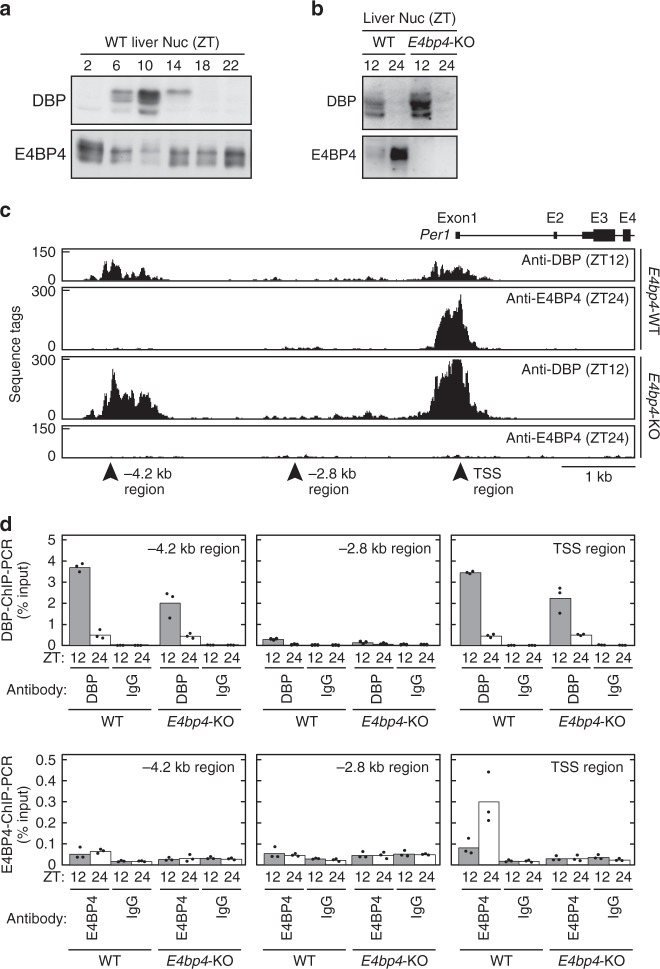
DBP-ChIP and E4BP4-ChIP analyses in mouse liver. **a**, **b** Anti-phasic expression of DBP and E4BP4 proteins. Liver nuclear extracts were prepared from *E4bp4*-KO and control mice at indicated time points, and were subjected to immunoblot analysis by using anti-DBP and anti-E4BP4 antibodies. Zeitgeber time (ZT) 0 and ZT12 correspond to the lights-on time and the lights-off time in LD cycles, respectively. Full images of the blots are shown in Supplementary Fig. 8. Data shown are the representative from three independent experiments with similar results. **c** ChIP-Seq analysis by using anti-DBP or anti-E4BP4 in the *Per1* gene locus. ChIP samples were prepared at ZT12 and ZT24 from *E4bp4*-KO and control mice, and were subjected to deep sequencing. TSS refers to transcription start site. **d** The ChIP samples were subjected to ChIP-PCR analysis with primer sets that amplify DNA regions indicated by the arrowheads in **c**. Anti-rhodopsin antibody 1D4 was used as a control IgG. Bars and dots represent means and individual data (*n* = 3), respectively. In this study, the number *n* is equal to the number of mice used for each sample at each time point

In this study, we aimed to determine the functional D-box sequences and explore in vivo roles of D-box-mediated transcriptional regulation. For biochemical analyses of DBP and E4BP4 proteins, we generated specific antibodies against these proteins. The antibodies detected rhythmic expression of DBP protein and anti-phasic expression rhythms of E4BP4 protein in the mouse liver (Fig. 1a, b, Supplementary Fig. 1a, b), as reported previously^5,9^. These antibodies were examined for efficiency of precipitation of a known D-box-containing DNA fragment in the *Per1* promoter region (Fig. 1c, TSS region)^7^. ChIP-PCR analysis showed that the DBP antibody precipitated the DNA fragment from mouse liver lysate prepared at ZT12, and the DBP-ChIP level was significantly reduced at ZT24 (Fig. 1d, TSS region; *p* = 8.7 × E-7, two-sided Student’s *t* test). On the other hand, the E4BP4-ChIP level in the TSS region was higher at ZT24 when compared with the level at ZT12, and the E4BP4-ChIP signals were almost abolished in the livers of *E4bp4*-knockout (KO) mice (Fig. 1d, TSS region). These ChIP signals were also reduced in a DNA region distant from the D-box sequence in the *Per1* gene locus (Fig. 1d, −2.8 kb region). Intriguingly, the rhythmic expression of DBP protein (Fig. 1b) and its rhythmic binding to the D-box (Fig. 1d, TSS region) were almost unaffected in *E4bp4*-KO mice, indicating that the anti-phasic DNA-binding of E4BP4 is dispensable for rhythmic recruitment of DBP to the D-box.

To develop genome-wide mapping of DBP-binding sites and E4BP4-binding sites, the ChIP DNA fragments were prepared from *E4bp4*-KO mouse and littermate control livers at ZT12 for the DBP-ChIP and at ZT24 for the E4BP4-ChIP. The ChIP samples were subjected to deep sequencing, which yielded 70–100 million tags in each sample (Supplementary Data 1). These tags were mapped onto the mouse genome, and the peak calling program MACS2 identified 6066 DBP-binding sites at ZT12 and 3064 E4BP4-binding sites at ZT24 in the control livers (Supplementary Data 1). The E4BP4-ChIP signals at the 3064 E4BP4-binding sites were significantly reduced in *E4bp4*-KO livers (wild type: 16.0 ± 12.4, *E4bp4*-KO: 1.8 ± 0.2, *p* = 1.3 × E-111, two-sided Student’s *t* test). Among the 3064 E4BP4-binding sites, DBP-binding signals were detected at 1490 sites, which we defined as DBP/E4BP4-common sites. When the 1490 common sites were compared with the previous E4BP4-ChIP data^10^, E4BP4-binding signals were detected at 1284 sites (1284/1490 = 86.2%), indicating high reliability of the current ChIP-Seq data. Typically, strong peaks of DBP-ChIP tags at ZT12 and E4BP4-ChIP tags at ZT24 were detected at the *Per1* TSS region (Fig. 1c), consistent with the ChIP-PCR analysis (Fig. 1d). Notably, our ChIP-Seq data identified a so-far unidentified DBP-binding site in the *Per1* −4.2 kb region, where no significant E4BP4-binding signal was detected (Fig. 1c, d; *p* = 0.49, two-sided Student’s *t* test). A similar DBP-preference site was detected in the intron 1 region (+2.7 kb region) of the *Per2* gene locus, in which a DBP/E4BP4-common site was located in the TSS region (Supplementary Fig. 1c, d).

### Functional D-box sequences defined by MOCCS2

**Fig. 2:**
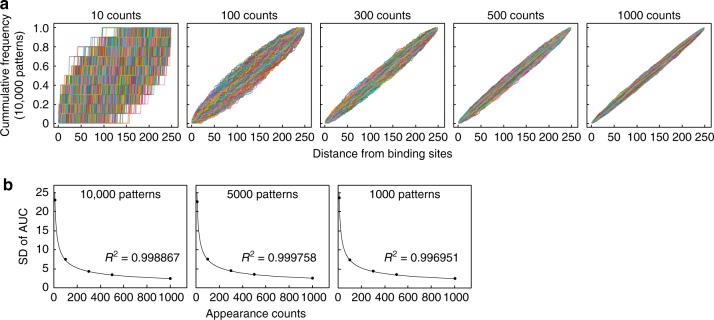
Simulation for determining standard deviations of AUC in MOCCS2. **a** Shown are 10,000 patterns of cumulative relative frequency curves calculated from random histograms with appearance counts indicated above individual graphs. **b** Standard deviations (SDs) of AUC calculated from indicated patterns of random histograms (*y* axis) were plotted against appearance counts (*x* axis), and well fitted with an exponential equation “*Y* = 71.303 × *X*^−0.5^”. See also Supplementary Fig. 2 for 5000 and 1000 patterns of the cumulative relative frequency curves

The palindromic sequence TTACGTAA and its one-mismatched sequence TTATGTAA were termed as the D-box (DBP/E4BP4-binding sequences)^3^. These sequences were identified from ten DBP/E4BP4-binding sites in selected clock genes (*Per1*, *Per2*, *Per3*, *Rev-erbα, Rev-erbβ*, *Rorα*, and *Rorβ*), and therefore previous estimates on D-box sequences may have been inaccurate. In a previous study, we developed a bioinformatics tool termed motif centrality analysis of ChIP-Seq (MOCCS), and extracted functional E-box motifs including noncanonical sequences from CLOCK-ChIP-Seq data^11,12^. This bioinformatics tool is based on the fact that DNA-binding sequences of transcription factors frequently appear at around their binding sites (peak positions of sequence tags) determined by ChIP-Seq. In MOCCS analysis, each DNA sequence was characterized by the area under the curve (AUC) that quantitatively represents sharpness of the histogram of its appearance around the binding sites^11^. AUC was calculated from area under the cumulative relative frequency curve in which cumulative appearance counts were plotted against the distance from the binding sites. MOCCS has a weak point in that some irrelevant sequences with low appearance counts are raised as positive motifs due to large standard deviations (SDs) of the AUC of the irrelevant sequences. To exclude such false positive sequences, we mathematically derived an equation that calculates SD of the AUC using the appearance count (see Methods): [SD of AUC] = 71.303 × [appearance count]^−0.5^. We also computationally calculated SDs of the AUCs of random histograms with various appearance counts (10, 100, 300, 500, and 1000 counts) by generating 1000, 5000, and 10,000 patterns of random histograms (Fig. 2a, Supplementary Fig. 2), and confirmed that the equation well fits the simulated data (Fig. 2b). Accordingly, in MOCCS version 2 (abbreviated as MOCCS2), the MOCCS2 score of each sequence was defined as a relative value of AUC normalized by the SD at its appearance count:

$${\mathrm{MOCCS2}\,{score} = \frac{{[AUC]}}{{71.303 \times [appearance\,count]^{ - 0.5}}}}$$

**Fig. 3:**
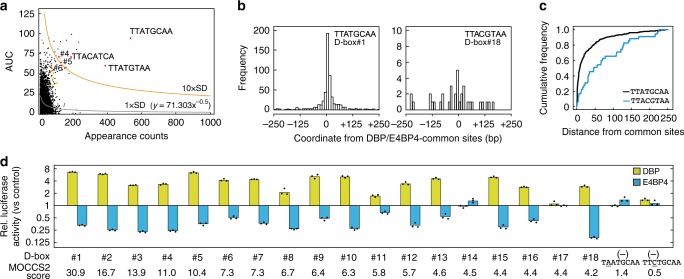
Identification of functional D-box sequences by MOCCS2. **a** MOCCS2 analysis of 1490 DBP/E4BP4-common sites. AUC of all 8-mer sequences (*y* axis) were plotted against their appearance counts (*x* axis). D-box#1-#6 are shown by red dots, D-box#7-#12 by orange, D-box#13-#18 by yellow, and others by black. Shown are also two exponential equations indicating 1 × SD (black line, *Y* = 71.303 × *X*^−0.5^) and 10 × SD (orange line, *Y* = 713.03 × *X*^−0.5^). **b** Frequency distribution of TTATGCAA (D-box#1) and TTACGTAA (D-box#18) around the DBP/E4BP4-common sites. The bin size of the *x* axis is 10 bp. **c** Cumulative relative frequency curves of D-box#1 and #18 around the common sites. Those of all the D-box#1-#18 sequences are shown in Supplementary Fig. 3b, c. **d** Dual luciferase reporter assays by using all the D-box#1-#18 sequences. The effects of DBP and E4BP4 on transcriptional activities were investigated by using luciferase reporters each harboring the indicated D-box or its related sequence. Ratios of bioluminescence signals from firefly luciferase relative to those from *Renilla* luciferase (internal control) for D-box reporters were normalized with the ratio for a control reporter (an empty vector, pGL3N). Fold changes of the normalized signal ratios relative to those in the absence of DBP and E4BP4 were shown in a semi-log plot. Bars and dots represent means and individual data (*n* = 3), respectively. See also Supplementary Fig. 3d

We then applied MOCCS2 to 1490 DBP/E4BP4-common sites, and calculated MOCCS2 scores for all 5-mer to 10-mer sequences (Supplementary Data 2). HOMER, a widely used tool for predicting DNA-binding motifs of transcription factors (http://biowhat.ucsd.edu/homer)^13^, revealed that at least 8-mer sequences are recognized by DBP and E4BP4 proteins (Supplementary Fig. 3a). Among 8-mer sequences in MOCCS2 analysis, TTATGCAA (termed D-box#1) was identified as having the highest MOCCS2 score (30.9) (Table 1, Supplementary Data 2, Fig. 3a), consistent with a sharp histogram of its appearance around the binding sites (Fig. 3b, Supplementary Fig. 3b) and strong convexity of the cumulative relative frequency curve (Fig. 3c, Supplementary Fig. 3c). This sequence is known to play a regulatory role in the *Per1* TSS region (Fig. 1c)^3,5,7^, but it is not included in the previously defined D-box motif TTAYGTAA^3^. When slip sequences of TTATGCAA such as TATGCAAN and NTTATGCA were eliminated, the second sequence was TTATGTAA (D-box#2), which is a well-established D-box motif found in the promoter region of the *Per2* gene^3^. TGATGTAA (D-box#3), TTATGTCA (D-box#4), and TTGTGTAA (D-box#5) are two-mismatched sequences of D-box#1 or alternatively are considered one-mismatched sequences of D-box#2. TTATACAA (D-box#6) is a one-mismatched sequence of D-box#1. We found that TTACGTAA (D-box#18) (Table 1, Fig. 3b, c) is the second twin of the previously defined D-box motif, TTAYGTAA^3^. In order to evaluate the MOCCS2 result, we prepared eighteen plasmids, each of which harbored a triple tandem repeat of one of D-box#1 to #18 sequences. Dual luciferase reporter assays in HEK293T cells showed that DBP activated and E4BP4 repressed promoter activities though the D-box sequences with at least the 10 highest MOCCS2 scores (Fig. 3d, Supplementary Fig. 3d). In contrast, only marginal effects of DBP and E4BP4 on the promoter activity were observed by using one-mismatched sequences of D-box#1 with low MOCCS2 scores, such as TTCTGCAA (0.5) and TAATGCAA (1.4) (Fig. 3d, Supplementary Fig. 3d). These data supported our conclusion that functional DNA sequences can be comprehensively defined by the improved bioinformatics method MOCCS2 from ChIP-Seq data.

**Table 1:** List of D-box motif sequences revealed by MOCCS2

Rank	Sequence	AUC	Appearance count	MOCCS2 score
D-box#1	**TTATGCAA**	94.0	537	30.9
D-box#2	**TTATG** **T** **AA**	60.4	390	16.7
D-box#3	**T** **G** **ATG** **T** **AA**	71.8	189	13.9
D-box#4	**TTATG** **TC** **A**	71.8	119	11.0
D-box#5	**TT** **G** **TG** **T** **AA**	64.7	132	10.4
D-box#6	**TTAT** **A** **CAA**	60.5	75	7.3
D-box#7	**TTA** **G** **G** **T** **AA**	60.1	74	7.3
D-box#8	**T** **G** **ATGCAA**	51.2	87	6.7
D-box#9	**TTAT** **CT** **AA**	53.5	73	6.4
D-box#10	**TTA** **C** **GCAA**	92.3	24	6.3
D-box#11	**T** **GC** **TG** **T** **AA**	45.1	85	5.8
D-box#12	**TTATG** **A** **AA**	39.0	107	5.7
D-box#13	**TT** **G** **TGCAA**	36.5	81	4.6
D-box#14	**TT** **TG** **GCAA**	42.0	58	4.5
D-box#15	**TTAT** **AT** **AA**	37.6	71	4.4
D-box#16	**TTA** **CT** **CAA**	39.5	63	4.4
D-box#17	**TTAT** **TGC** **A**	42.0	55	4.4
D-box#18	**TTA** **C** **G** **T** **AA**	50.7	35	4.2

### Perturbation of circadian output by *E4bp4* deficiency

**Fig. 4:**
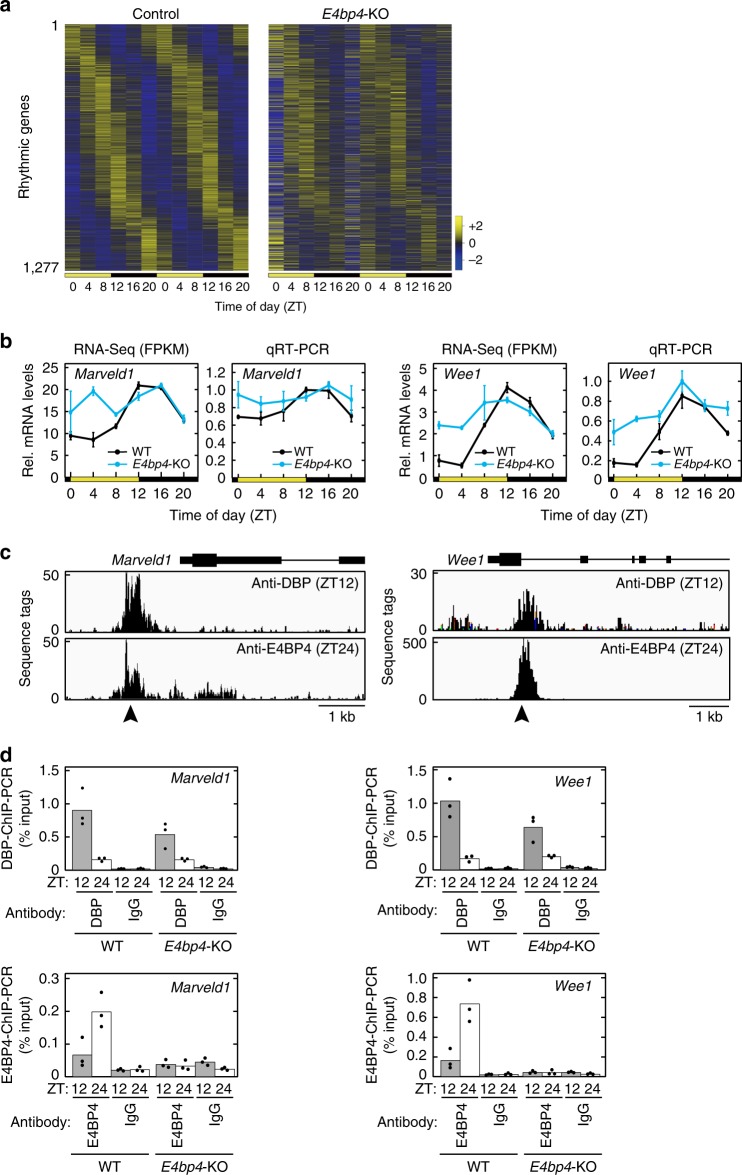
Dysfunction of circadian output in *E4bp4*-KO liver. **a** Heat maps of mRNA levels of 1277 rhythmic genes determined by the RNA-Seq. Genes were ordered by their peak phases in control mice from early day to late night. The FPKM values were normalized so that the mean and the variance were set to 0 and 3, respectively, for each row of the maps. **b** Temporal profiles of mRNA levels of representative target genes in livers of *E4bp4*-KO and control mice, determined by RNA-Seq and qRT-PCR analyses. Data are shown as means with SD (*n* = 2) for RNA-Seq and means with SEM (n = 3) for qRT-PCR. **c** DBP-ChIP-Seq and E4BP4-ChIP-Seq data in the representative target gene loci. **d** DBP-ChIP and E4BP4-ChIP samples were subjected to ChIP-PCR analysis with primer sets that amplify the DNA region indicated by arrowheads in **c**. Bars and dots represent means and individual data (*n* = 3), respectively

ChIP-Seq and MOCCS2 analyses revealed functional D-box sequences, so we then focused on physiological roles of D-box-mediated transcriptional regulation. In mammalian circadian clockwork, DBP and E4BP4 regulate transcription by their anti-phasic binding to D-box^5^ and contribute to determining the circadian phase of mRNA rhythms^3,8^. We investigated the effects of *E4bp4* deficiency on gene expression rhythms in the mouse liver. The poly(A)-tailed RNAs were prepared from *E4bp4*-KO livers isolated at biologically duplicated 6 time points in 12-h light:12-h dark (LD) condition, and were subjected to deep sequencing. The sequence tags were mapped onto the mouse genome, and this analysis yielded ~40 million mapped tags for each sample (Supplementary Data 3). Among 54,733 mouse genes including non-coding RNAs (Ensembl, release 95), 12,758 genes were expressed, and 1277 genes (10.0% of the expressed genes) were found to be rhythmic (*p* < 0.05, JTK cycle algorithm) in the control mice (Supplementary Data 3). The heat map of the 1277 rhythmic genes showed a great diversity in their circadian phases (Fig. 4a), indicating cooperative actions of the E-box, D-box, and RRE in circadian gene expressions as previously reported^3,8^. It is remarkable that the temporal expression profiles of the rhythmic genes were markedly affected by *E4bp4* deficiency (Fig. 4a, Supplementary Data 3), indicating that *E4bp4* is important for normal circadian output.

Among the 1277 rhythmic genes, 359 genes were *E4bp4*-dependent rhythmic genes that showed robust expression rhythms (*p* < 0.01) in the control but lost their rhythmicities (*p* ≥ 0.05) in the *E4bp4*-KO livers (Supplementary Fig. 4a). Among the *E4bp4*-dependent rhythmic genes, *Marveld1* and *Wee1* showed expression peaks at ZT12-16, whereas their expression levels were constantly high in the *E4bp4*-KO livers (Fig. 4b). These data are consistent with the expression rhythm of the E4BP4 repressor peaking at ZT0-2 (Fig. 1a, b). ChIP-Seq and ChIP-PCR analyses demonstrated rhythmic binding of DBP and E4BP4 to the promoter region of *Marveld1* and to the intronic region of *Wee1* (Fig. 4c, d). It is noted that D-box#2 (TTATGTAA) was found at around the DBP/E4BP4-common sites in the *Marveld1* and *Wee1* gene loci. Gene ontology (GO) analysis showed that the *E4bp4*-dependent rhythmic genes were enriched with genes involved in metabolic pathways (Supplementary Fig. 4b). It has also been reported that *E4bp4*-KO mice suffer from inflammatory diseases of the intestine^14–16^. The current study will provide clues to understanding *E4bp4*-mediated transcriptional regulation in other physiological processes.

### Acute induction of *E4bp4* expression for circadian input

**Fig. 5:**
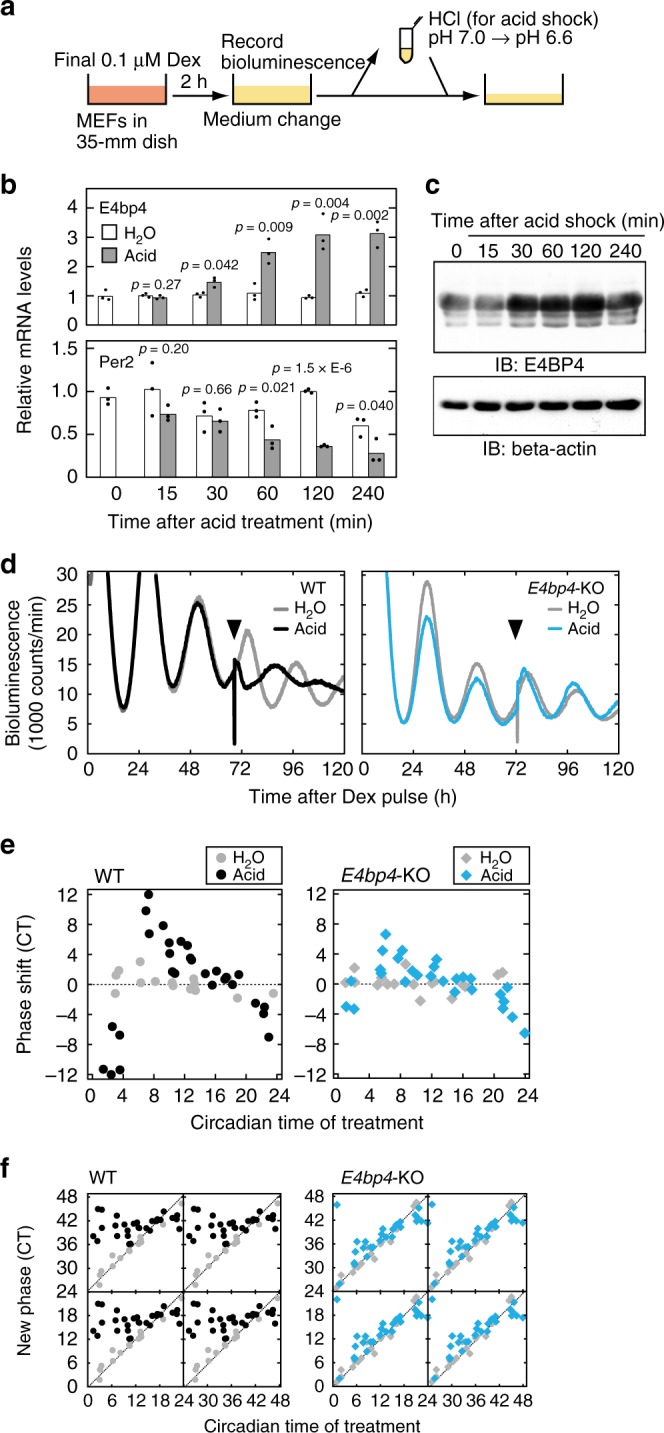
Essential role of E4BP4 for acid-evoked phase resetting. **a** For acid treatment, pHo was changed from 7.0 to 6.6 by adding 1M HCl solution to the culture media. In control experiments, the same volume of water was added (H_2_O). **b** Changes of *E4bp4* and *Per2* mRNA levels in response to the acid treatment. MEFs collected at the indicated time after the acid treatment were subjected to qRT-PCR analysis. The signal values for each mRNA were normalized to those of Rps29 mRNA (internal control) and the mean value at time 0 was set to 1. Bars and dots represent means and individual data (*n* = 3), respectively. The indicated *p* values were calculated by two-sided Student’s *t* test versus H_2_O. **c** Induction of E4BP4 protein in response to the acid treatment. MEFs collected at the indicated time after the acid treatment were subjected to immunoblot analysis with the indicated antibodies. Full images of the blots are shown in Supplementary Fig. 8. Data shown are the representative from three independent experiments with similar results. **d** Phase shifts of bioluminescence rhythms in the PER2::LUC MEFs (WT) or PER2::LUC/*E4bp4*-KO MEFs (*E4bp4*-KO) in response to the acid treatment. The timing of the acid treatment was indicated by arrowheads. **e**, **f** Phase response curves (PRCs) and phase transition curves (PTCs) of the cellular rhythms in response to the acid treatment in the control PER2::LUC MEFs (WT) or PER2::LUC/*E4bp4*-KO MEFs (*E4bp4*-KO)

E4BP4 protein levels were markedly upregulated in response to extracellular stimuli such as interleukin 3^17^, glutamate, H_2_O_2_^18^, and insulin^19^. DBP and E4BP4 competitively bind to the D-box^5^, and therefore acute E4BP4 induction might interfere with DBP-binding to the D-box, leading to a phase shift of the circadian clock. To examine the possibility that E4BP4 induction is a key step for circadian phase control, we searched for a specific stimulus that increases E4BP4 protein levels and causes phase shifts of the circadian clock. We previously demonstrated that alkalization or acidification of extracellular pH (pHo) reset the cellular clock in cultured rat-1 fibroblasts^20^. The alkalization triggered activation of extracellular TGF-beta that stimulated the ALK-SMAD3 pathway and induced *Dec1*, which reset the clock^20^. In contrast, the molecular mechanism of this acidification-evoked phase resetting has remained uncharacterized. Here we found that *E4bp4* mRNA was immediately upregulated in mouse embryonic fibroblasts (MEFs) one hour after pHo was shifted from 7.0 to 6.6 by adding HCl to the cultured media (Fig. 5a, b, Supplementary Fig. 5a). Protein levels of E4BP4 were also elevated after the acidification (Fig. 5c), whereas mRNA levels of *Per2* declined gradually (Fig. 5b). The acid-induced response of *E4bp4* mRNA was not suppressed by treatment with cycloheximide, a protein synthesis inhibitor, but abrogated by actinomycin D, a transcription inhibitor (Supplementary Fig. 5b), suggesting an immediate early response of *E4bp4* to the acid treatment.

To examine whether E4BP4 induction is required for the acid-evoked phase resetting, we isolated *E4bp4*-KO/PER2::Luc MEFs, in which circadian rhythms can be examined by real-time monitoring of the bioluminescence from Luciferase fused with endogenous PER2 protein. In the control PER2::Luc MEFs, we observed apparent phase shifts, when pHo of the cultured media was shifted from 7.0 to 6.6 (Fig. 5a) several hours after the trough time of the bioluminescence rhythms (Fig. 5d), as was previously observed in rat-1 fibroblasts^20^. On the other hand, faint phase shifts were induced by the acidification several hours after the peak time (Supplementary Fig. 5c). Phase response curves and phase transition curves showed phase-dependent phase shifts and revealed type-0 resetting of cellular rhythms in response to the acidification, respectively (Fig. 5e, f, left). Importantly, the acidification-induced phase shifts were almost completely blocked in the *E4bp4*-KO/PER2::Luc MEFs (Fig. 5d–f, right), indicating an essential role of the acute induction of E4BP4 in acid-evoked phase resetting. The present work revealed physiological roles of D-box-mediated transcriptional regulation, which is important for non-photic phase control in the peripheral clock.

## Discussion

In the present study, we generated anti-DBP and anti-E4BP4 antibodies, which enabled us to determine DNA regions recognized by DBP and E4BP4 proteins in a genome-wide manner (Fig. 1, Supplementary Data 1). In general, ChIP-Seq analysis provides information about genomic DNA regions recognized by a transcription factor, and many bioinformatics tools such as MEME and HOMER have been employed to extract a representative DNA-binding motif from ChIP-Seq data. However, a bioinformatics tool that can determine all DNA-binding sequences of a transcription factor is required. In a previous study, we developed a bioinformatics tool termed MOCCS to provide a comprehensive list of DNA-binding sequences of a transcription factor^11,12^. In the present study, we improved this tool by calculating a new parameter, the MOCCS2 score, which represents the significance of appearance frequency of a sequence around binding sites of a transcription factor (Fig. 2). The original version of MOCCS raised eight candidates for CLOCK-binding sequences (Supplementary Fig. 6), among which TACGTA having the 7th highest AUC but a very low appearance count was considered a false positive sequence because it had no significant activity in promoter assay^11^. When MOCCS2 was applied to the previous CLOCK-ChIP-Seq data, the MOCCS2 score of TACGTA (6.5) was obviously lower than those of the other candidates (102.8–21.6) (Supplementary Fig. 6). It is clear that MOCCS2 analysis excludes such false positive sequences. In the present study, functional D-box sequences were identified by MOCCS2 analysis of 1490 sites that are recognized commonly by DBP and E4BP4 (Fig. 3, Table 1). On the other hand, our ChIP-Seq analysis identified 4573 DBP-binding sites where no significant binding of E4BP4 was detected (Supplementary Data 1). In MOCCS2 analysis of the 4573 sites, TTACCCAA, a two-mismatched sequence of D-box#1, showed a higher MOCCS2 score (10.6) (Supplementary Data 4), contrasting its lower score (3.0) in MOCCS2 analysis of the 1490 DBP/E4BP4-common sites (Supplementary Data 1). It should be noted that TTACCCAA was found in regions with DBP-preference sites such as the *Per1* −4.2 kb region (Fig. 1c) and the *Per2* +2.7 kb region (Supplementary Fig. 1). These results indicate that MOCCS2 is a powerful bioinformatics tool in determining all DNA-binding sequences from ChIP-Seq data.

In our previous study^11^, the ChIP-Score was defined as the total number of sequence tags that were mapped to all the CLOCK-binding sites within ±10 kb of the transcription start site of each gene or in the gene body. ChIP-Score analysis of the current ChIP-Seq identified 6696 genes as the targets of DBP and/or E4BP4 proteins (Supplementary Data 5). Among the 6696 DBP/E4BP4 targets, 3300 genes were judged as expressed based on the liver RNA-Seq data (Supplementary Data 3). Their ratio (3300/6696 = 49.3%) was 2.1-fold higher than the ratio of the number of the expressed genes to that of all genes (12,758/54,733 = 23.3%), suggesting that genes targeted by DBP and E4BP4 are more frequently expressed in the mouse liver. On the other hand, we found 359 *E4bp4*-dependent rhythmic genes that showed robust expression rhythms in the control but lost their rhythmicities in the *E4bp4*-KO liver (Supplementary Fig. 4b). Among the 359 genes, 130 genes were also included in the 3300 expressed DBP/E4BP4 targets. Their ratio (130/3300 = 3.94%) was 1.4-fold higher than the ratio of the number of the *E4bp4*-dependent rhythmic genes to that of the expressed genes (359/12,758 = 2.81%), indicating that genes targeted by DBP and E4BP4 become more frequently arrhythmic in the *E4bp4*-KO livers.

In *Drosophila*, an *E4bp4* homolog *vrille* rhythmically represses transcription of *dclock* gene and serves as a key component of the core circadian oscillation^21–23^. It was also reported that siRNA-mediated knockdown of *E4bp4* lengthened the circadian period in cultured rat-1 cells^24^. However, our RNA-Seq analysis showed that *E4bp4* deficiency resulted in no remarkable changes of expression profiles of the core clock genes (Supplementary Fig. 7a, b). The normal circadian oscillation was also confirmed by monitoring the wheel-running rhythms of *E4bp4*-KO mice under constant dark conditions (Supplementary Fig. 7c, d; WT: 23.85 ± 0.19 hr, KO: 23.76 ± 0.23 hr). To our knowledge, this is the first report showing that the *E4bp4* gene is dispensable for maintaining circadian rhythms of mouse locomotor activities. In contrast to the marginal effect on rhythmic expression of core clock genes (Supplementary Fig. 7a, b), *E4bp4* deficiency caused dysregulation of circadian output genes (Fig. 4, Supplementary Fig. 4). Previously, it was reported that locomotor activity rhythms were almost intact in triple KO mice of PAR bZip factors^25^, whereas these deficiencies caused strong effects on circadian outputs^26^. DBP single-KO mice showed a shorter free-running period^27^, contrasting with the longer period phenotype of TEF or HLF single-KO mice (mentioned in the text of ref. ^25^). These results indicate that the importance of the D-box-mediated transcriptional regulation in mRNA rhythms is diverged among rhythmically expressed genes.

In addition to the role of the D-box in circadian clock outputs, we described an indispensable role of the *E4bp4* gene as an input to the clock (Fig. 5). *E4bp4* expression is induced in response to various extracellular stimuli such as interleukin 3^17^, glutamate, H_2_O_2_^18^, insulin^19^ (Supplementary Fig. 5a), and acidification of the culture media (Fig. 5b, c). It is not clear whether the acid-induced circadian phase shift has a physiological significance in vivo, but circadian rhythms are phase-shifted by physiological activities such as exercise and feeding^28^, which activate glycolysis leading to lactic acid accumulation. The acute E4BP4 induction at a time when its expression level is low could competitively interfere with DNA-binding of the PAR bZip factors to D-box. This should lead to a phase shift of the circadian clock. Intraperitoneal injection of insulin caused acute E4BP4 induction in mouse liver (Supplementary Fig. 5d), and thereby elevated its binding to the D-box located in the *Per1* and *Per2* promoter regions (Supplementary Fig. 5e). It was reported that insulin also induces *Per2* expression^29^ together with its repressor *E4bp4* expression (Supplementary Fig. 5d, e), and such feedback action of *E4bp4* may be important for transient response of *Per2* expression to insulin. In this study, we demonstrated that the acute induction of E4BP4 protein is essential for the type-0 resetting of the cellular rhythms elicited by acidification of the cultured media (Fig. 5d–f). In previous studies on the chicken pineal clock, we showed that light-dependent activation of sterol regulatory element-binding protein (SREBP) transcription factor remarkably elevated *E4bp4* mRNA levels, which led to suppression of *Per2* transcription and phase shifts^30–32^. Intriguingly, acidification of extracellular pH (to 6.8) triggers activation of SREBP in cancer cells^33^, suggesting a potential relationship between the acid-inducible *E4bp4* and the acidic microenvironment of tumors. It was also reported that D-box-mediated transcription is important for light-dependent induction of z*Per2* in zebrafish^34,35^. In mice, however, *E4bp4* deficiency had no significant effect on light-dependent phase shifts (Supplementary Fig. 7e), and E4BP4 has a pivotal role in non-photic phase control of the peripheral clocks. Collectively, we conclude that transcriptional regulation via D-box sequences plays key roles in the circadian inputs and outputs.

## Methods

### Animals

The animal experiments were approved by the animal ethics committee of the University of Tokyo. C57BL/6J mice were individually housed in cages with free access to food and water. *E4bp4-*KO mice were kind gifts from A. Thomas Look (Children’s Hospital Boston, Harvard Medical School). In the *E4bp4-*KO mice, the exon 2 of *E4bp4* gene was replaced by a neomycin cassette, as previously described^36^. Mice were reared in 12-h light:12-h dark cycles in a light-tight chamber at a constant temperature (23 ± 1 °C). PER2::LUC knock-in mice^37^ were used for monitoring bioluminescence rhythms. Wheel-running activity rhythms were monitored and analyzed with Clocklab software (Actimetrics) developed on MatLab (Mathworks), as previously described^38^.

### Antibodies for immunoblot and ChIP analyses

We generated anti-DBP and anti-E4BP4 antibodies in rabbits, now commercially available as anti-DBP (MBL, PM079) and anti-E4BP4 antibodies (MBL, PM097). We also used anti-rhodopsin 1D4^39^, anti-TBP (Santa Cruz Biotechnology, sc-421) and anti-beta-actin (Sigma, A2228). In immunoblot analysis, the bound primary antibodies were detected by horseradish peroxidase-conjugated anti-rabbit or anti-mouse IgG antibody (Kirkegaard & Perry Laboratories).

### Preparation of nuclear proteins

The nuclear proteins were isolated as previously described^40,41^. Mouse tissue (1 g, wet weight) was washed with ice-cold PBS and homogenized at 4 °C with 9 ml of ice-cold buffer A (10 mM HEPES-NaOH, 10 mM KCl, 0.1 mM EDTA, 1 mM dithiothreitol (DTT), 1 mM phenylmethylsulfonyl fluoride (PMSF), 4 μg/ml aprotinin, 4 μg/ml leupeptin, 50 mM NaF, and 1 mM Na_3_VO_4_; pH 7.8). The homogenate was centrifuged twice (700 × *g*, 5 min each), and the precipitate was resuspended in 2 ml of ice-cold buffer C (20 mM HEPES-NaOH, 400 mM NaCl, 1 mM EDTA, 5 mM MgCl_2_, 2% glycerol, 1 mM DTT, 1 mM PMSF, 4 µg/ml aprotinin, 4 µg/ml leupeptin, 50 mM NaF, and 1 mM Na_3_VO_4_; pH 7.8). After gentle mixing at 4 °C for 30 min, the suspension was centrifuged twice (21,600 × *g*, 30 min each), and the final supernatant was used as the “nuclear extract”.

### Chromatin immunoprecipitation

ChIP analysis was prepared as described previously^11^ with minor modifications. Livers were isolated at two time points, ZT12 and ZT24 (*n* = 3), from *E4bp4*-KO and the WT littermate mice. They were rinsed with ice-cold PBS and were homogenized with ice-cold buffer A (10 mM HEPES-NaOH, 10 mM KCl, 0.1 mM EDTA, 1 mM DTT, 1 mM PMSF, 4 μg/ml aprotinin, 4 μg/ml leupeptin, 50 mM NaF, and 1 mM Na_3_VO_4_; pH 7.8). The homogenate was centrifuged twice (700 × *g*, 5 min each), and the precipitate (nuclear fraction) was cross-linked by 1% formaldehyde in buffer A for 10 min at 25 °C. The cross-linking reaction was stopped by addition of 125 mM glycine (final concentration). The sample was then centrifuged (700 × *g*, 5 min), and the nuclei pellet was washed twice with buffer A and resuspended in IPB2 buffer (20 mM HEPES-NaOH, 137 mM NaCl, 1 mM EDTA, 5% glycerol, 1% Triton X-100, 1.67 mM MgCl_2_, 1 mM DTT, 1 mM PMSF, 4 μg/ml aprotinin, 4 μg/ml leupeptin, 50 mM NaF, and 1 mM Na_3_VO_4_; pH 7.8) supplemented with 1% SDS. The sample was then sonicated 16 times for 20 s each at intervals of 40 s (Branson Sonifier 450; set at 50% duty cycle, five output). The supernatant was diluted in IPB2 (final 0.1% SDS), and snap-frozen in liquid nitrogen. After thawing, the sample was centrifuged at 20,000 × *g* for 10 min at 4 °C, and the supernatant was incubated with anti-DBP antibody, anti-E4BP4 antibody, or an irrelevant antibody 1D4 while being gently rotated for 2 h at 4 °C. Protein G-coupled magnetic beads (Dynabeads, Dynal) were added to the mixture, followed by gentle rotation for 1 h at 4 °C. The beads were washed sequentially with the following buffers by using DynaMag-2 magnet: (i) IPB2 buffer; (ii) IPB2 buffer supplemented with 500 mM NaCl; (iii) TE buffer (10 mM Tris-HCl, 1 mM EDTA; pH 8.0) supplemented with 0.25 M LiCl, 1% NP-40, and 1% deoxycholate; and (iv) TE buffer. Finally, the beads were treated with 500 µl of the elution buffer (1% SDS, 0.1 M NaHCO_3_) and gently rotated for 30 min at room temperature, and the eluate was mixed with 20 µl of 5 M NaCl and incubated overnight at 65 °C. The de-cross-linked sample was then mixed with 10 μl of 0.5 M EDTA, 20 μl of 1 M Tris-HCl (pH 6.5) and 2 μl of 10 mg/ml Proteinase K, and the mixture was incubated for 2 h at 45 °C. The DNA was purified by extraction with phenol-chloroform-isoamyl alcohol (25:24:1) and subjected to ethanol precipitation. The final precipitate was used as the ChIP sample.

### ChIP-Seq analysis

The DBP-ChIP (at ZT12, *n* = 2) and E4BP4-ChIP samples (at ZT24, *n* = 2) prepared from *E4bp4*-KO mice and the WT littermates were sequenced on a HiSeq 3000 sequencer (36 bp, single end). The input samples prior to the immunoprecipitation were also subjected to the deep sequencing as controls. The sequence tags were mapped to the mouse genome by using Bowtie (v1.2.1) with parameter setting of “−a –best –strata -m 1 −p 4”^42^. BAM files of biological duplicates were merged using the “samtools merge” command. Peak calling was performed for the merged ChIP samples versus the merged input using MACS2 (v2.1.1) with default parameters^43^. The mapped tags were visualized by using Integrative Genomics Viewer^44^.

### MOCCS2 analysis

The equation that calculates [SD of AUC] from [appearance counts] was mathematically derived as follows. Let *W* be the size of the analyzed window where *k*-mer sequences are sought at around ChIP-peak positions. If a *k*-mer sequence appears only once at a random position within the window, its coordinate follows the uniform distribution U(0, *W*), whose variance is known to be *W*^2^/12. Because (i) AUC is calculated by subtracting *W*/2 from the coordinate and (ii) constant subtraction does not affect variance of probability distributions, variance of AUC is also *W*^2^/12 if the appearance count is 1. Next, assume that a *k*-mer sequence appears *C* times at random positions within the window. The variance of the sum of their coordinates becomes *CW*^2^/12, because variance of sum of random variables that follow the same probability distribution is proportional to the numbers of the variables. Then, because AUC is calculated by dividing the sum of their coordinates by *C* and subtracting *W*/2, the variance of AUC is (*CW*^2^/12)/*C*^2^ = *W*^2^/12 *C*, if the appearance count is *C*. Finally, we obtain [SD of AUC] by taking the square root of the variance:

$$[{\mathrm{SD}}\, {\mathrm{of}}\, {\mathrm{AUC}}] = \frac{[{\mathrm{window}}\, ({\mathrm{W}})]}{{\sqrt{12}} \times {\sqrt {[{\mathrm{appearance}}\, {\mathrm{count}}\, ({\mathrm{C}})]}}}$$

In MOCCS version 2 (abbreviated as MOCCS2), the “MOCCS2 score” of each sequence was defined as a relative value of AUC normalized by the SD at its appearance count:

$${\mathrm{MOCCS2}}\, {\mathrm{score}} = \frac{\mathrm{[AUC]}}{{[{\mathrm{SD}}\, {\mathrm{of}}\, {\mathrm{AUC}}]}}$$

In this study, *W* was set to 250 + 1 – (*k*/2), because *k*-mer sequences do not appear at the end of the 250-bp windows. If *k* = 8, [SD of AUC] was 71.303/*C*^0.5^, as shown in the Result section. If *k* = 6, [SD of AUC] was 71.591/*C*^0.5^. The MOCCS2 is freely available via https://github.com/yuifu/moccs.

### RNA preparation and RNA-Seq analysis

RNA-Seq analysis was performed as previously described^45^ with minor modifications. The total RNA was prepared from livers of *E4bp4*-KO/PER2::Luc mice and the littermate PER2::Luc mice at six time points throughout the day (ZT0, 4, 8, 12, 16, and 20; *n* = 2) by using the TRIzol reagent (Invitrogen) and RNeasy mini kit (QIAGEN) according to the manufacturer’s protocol. Poly(A)-tailed RNA was isolated from the total RNA as the manufacturer’s protocol, and was sequenced on a HiSeq 3000 (36 bp, single end). The mouse genome sequence was obtained from UCSC Genome Browser (mm10, http://genome.ucsc.edu/). The annotated gene models (GRCm38) were taken from Ensembl (release 95, http://www.ensembl.org/). Hisat2 (v2.1.0) was used for mapping RNA-Seq data with parameter setting of “-p 4 —dta -q -x”^46^. The expression level of each gene was quantified as fragments per kilobase of exon per million fragments (FPKM) by using both StringTie (v1.3.4) with parameter setting of “-e -G”^47^ and Ballgown (v2.12.0) with default parameters^48^. A gene was defined as “expressed” if the average of FPKM values of the 12 samples (6 time points, duplicate) in *E4bp4*-KO or control mice was higher than 1.0. A gene was defined as “rhythmic” if JTK cycle program^49^ detected any circadian rhythmicity with *p* < 0.05.

### Quantitative PCR

For quantitative PCR (qPCR) analysis, the ChIP samples were subjected to real-time PCR (Applied Biosystems) using GoTaq Master Mix (Promega) with gene-specific primers (Supplementary Table 1). For qRT-PCR, the total RNA samples prepared at ZT0, 4, 8, 12, 16, and 20 were reverse transcribed by Go Script Reverse Transcriptase (Promega) with both an anchored (dT)15 primer and a random oligo primer. The cDNA samples were subjected to the qPCR analysis with gene-specific primers (Supplementary Table 1).

### Dual luciferase reporter assay

HEK293T17 cells in 24-well plates were transiently transfected by using polyethylenimine (Polysciences, #24765) with 100 ng Flag-DBP/pSG5 or Flag-E4BP4/pSG5 in combination with 10 ng of firefly luciferase reporter plasmids and 0.5 ng of a *Renilla* luciferase plasmid (pRL-SV40) as an internal control. The total amount of DNA was adjusted to 410.5 ng by adding the empty expression plasmid pSG5. A triple tandem repeat of one of D-box sequences was inserted into a BglII site of a firefly luciferase reporter plasmid (pGL3N) as previously described^11^. The inserted sequences were shown in Supplementary Table 1. The transfected cells were collected 36 h after the transfection and subjected to the dual luciferase assay according to the manufacturer’s protocol with the aid of a fluorescence plate reader (Promega GloMax). Internal control was used to normalize the transfection efficiency.

### Real-time monitoring of cellular rhythms and acidification

Cellular bioluminescence rhythms were monitored as described previously^50^ with minor modifications. In brief, MEFs were prepared from PER2::LUC knock-in mice^37^. The MEFs were maintained at 37 °C under 5% CO_2_, 95% air in Dulbecco’s modified Eagle’s medium (SIGMA) supplemented with 25 units/ml penicillin, 25 µg/ml streptomycin, and 10% fetal bovine serum. PER2::Luc MEFs were plated on 35-mm dishes (1.0 × 10^6^ cells/dish) and cultured at 37 °C under 5% CO_2_. After 24 hr, the cells were treated with 0.1 µM (final) dexamethasone (Dex) for 2 h, and then the media were replaced by a recording media (phenol-red free Dulbecco’s modified Eagle’s medium (SIGMA) supplemented with 10% fetal bovine serum, 3.5 g/l glucose, 25 U/ml penicillin, 25 µg/ml streptomycin, 0.1 mM luciferin, and 10 mM HEPES-NaOH; pH 7.0). The bioluminescence signals of the cultured cells were recorded continuously for 5–10 days at 37 °C in air with Dish Type Luminescencer, Kronos (Atto, AB-2500 or AB-2550) or LumiCycle (Actimetrics).

For acid treatment, extracellular pH (pHo) was shifted from 7.0 to 6.6 by adding a minimal volume of 1 M HCl solution to the cultured media as previously described^20^. In control experiments, the same volume of water was added (H_2_O). Circadian time (CT) 0 was defined as the time points of the troughs of the bioluminescence signal waveforms. The phase shifts were calculated from the time of peaks and troughs of the bioluminescence rhythms.

### Reporting summary

Further information on research design is available in the Nature Research Reporting Summary linked to this article.

## Supplementary information

Supplementary Information

Description of additional supplementary items

Reporting Summary

Supplementary Data 1

Supplementary Data 2

Supplementary Data 3

Supplementary Data 4

Supplementary Data 5

## Data Availability

Illumina sequencing data for the ChIP-Seq and the RNA-Seq are available in the DDBJ/EBI/NCBI databases under the accession numbers PRJDB7796 and PRJDB7789. Other data are available from the authors upon request.
