# Supplementary material for: Functional D-box sequences reset the circadian clock and drive mRNA rhythms
Source: Commun Biol. 2019 Aug 8;2:300. doi: 10.1038/s42003-019-0522-3 (PMC6687812; doi:10.1038/s42003-019-0522-3)
Supplement: Supplementary file 1 — Supplementary Information [file 42003_2019_522_MOESM1_ESM.pdf]

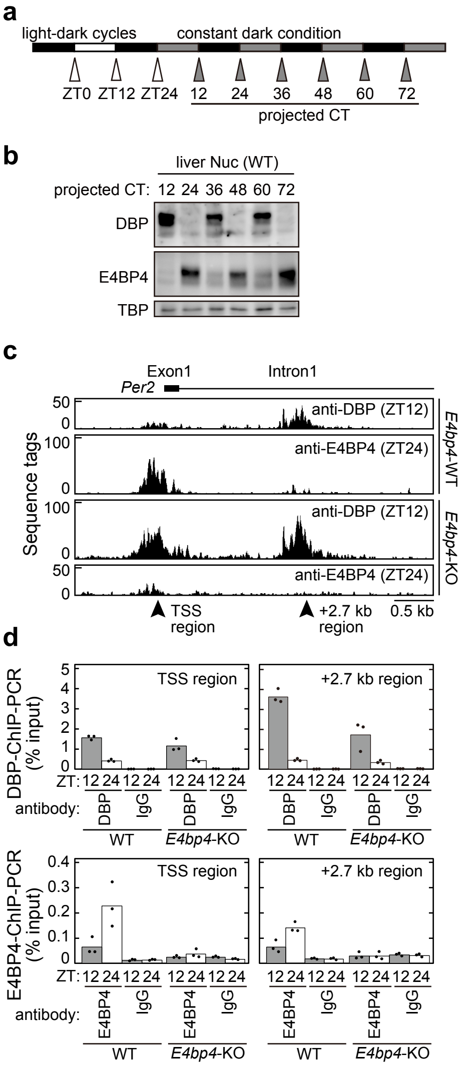

Supplementary Fig. 1 DBP-ChIP and E4BP4-ChIP analyses in the *Per2* gene locus

(a,b) Anti-phasic expression of DBP and E4BP4 proteins in constant dark condition. Liver nuclear extracts were prepared from WT mice at indicated time points, and were subjected to immunoblot analysis by using anti-DBP and anti-E4BP4 antibodies. TBP serves as a loading control. Full images of the blots are shown in Supplementary Fig. 8. (c) ChIP-Seq analysis by using anti-DBP or anti-E4BP4 in the *Per2* gene locus. ChIP samples were prepared at ZT12 and ZT24 from E4bp4-KO and control mice, and were subjected to deep sequencing. TSS refers to transcription start site. (d) The ChIP samples were subjected to ChIP-PCR analysis with primer sets that amplify DNA regions indicated by arrowheads in panel c. Anti-rhodopsin antibody 1D4 was used as a control IgG. Bars and dots represent means and individual data (n = 3), respectively.

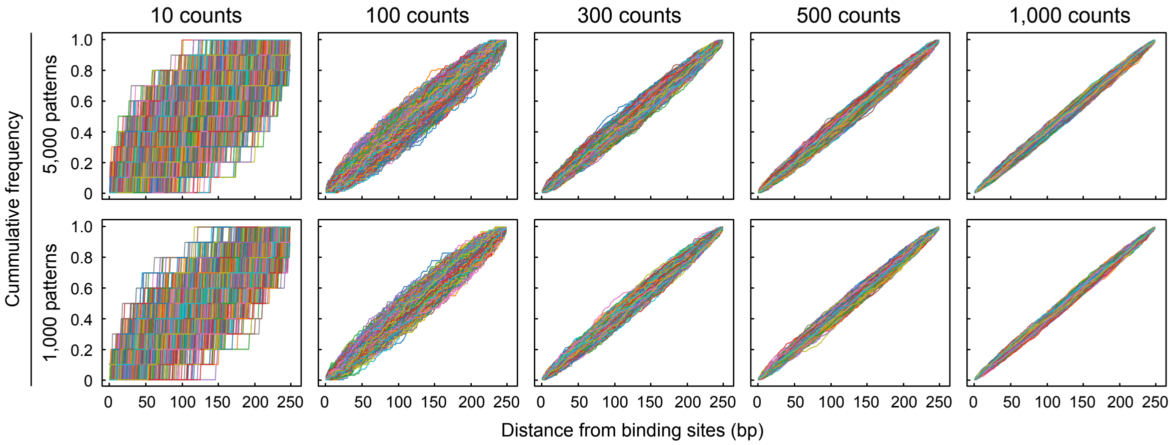

Supplementary Fig. 2 Simulation for determining standard deviations of AUC in MOCCS2

Shown are 5,000 (top) and 1,000 (bottom) patterns of cumulative relative frequency curves calculated from random histograms with indicated appearance counts. See also Fig. 2.

Supplementary Fig. 3

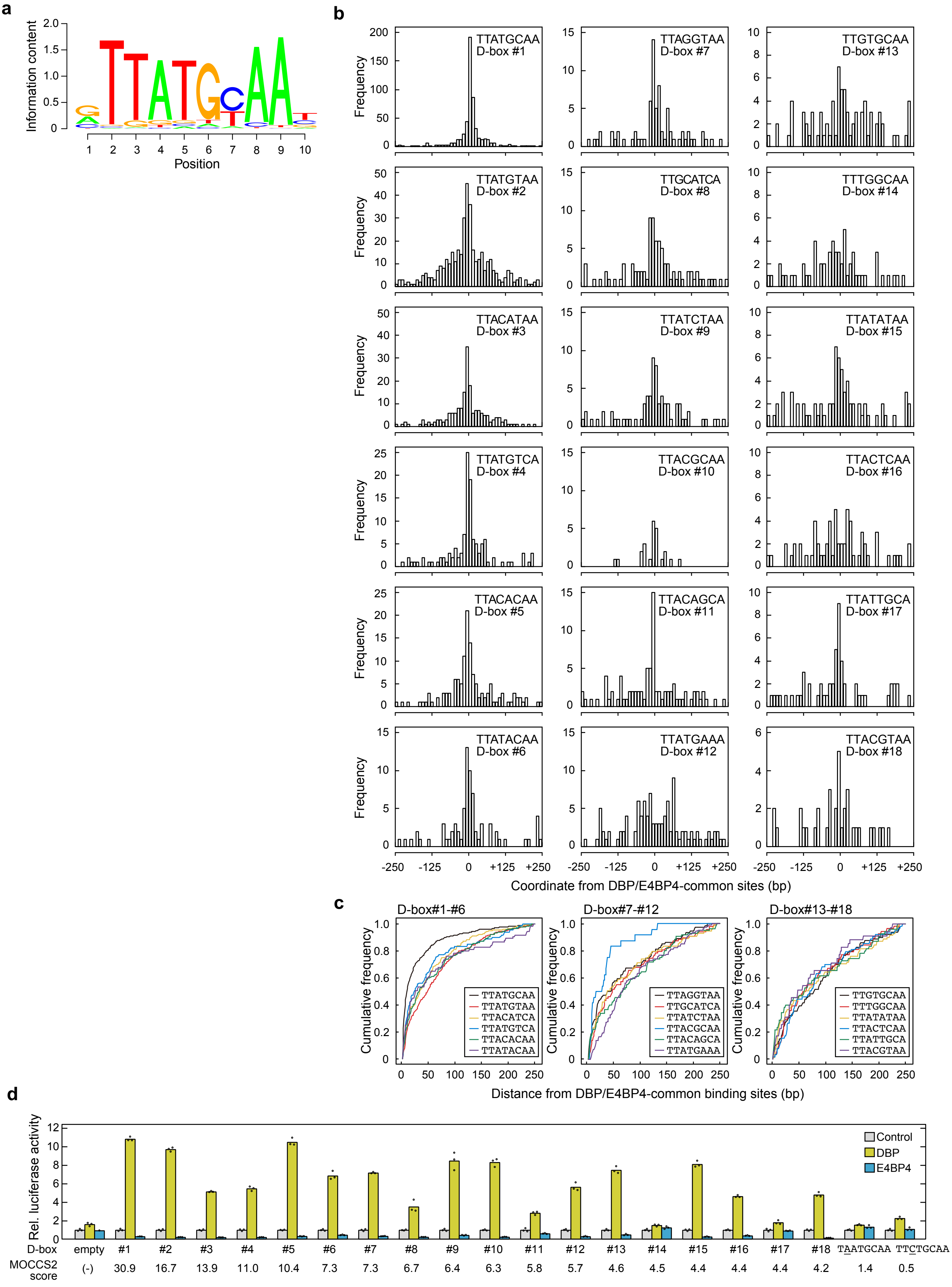

Supplementary Fig. 3 Identification of functional D-box sequences by MOCCS2

(a) Over-represented D-box sequences determined by HOMER. DNA sequences in the window of ±200 bp around DBP/E4BP4-common sites were used for the sequence analysis. (b) Frequency distribution of all the D-box#1-#18 sequences around 1,490 DBP/E4BP4-common sites. The bin size of x-axis is 10 bp. (c) Cumulative relative frequency curves of all the D-box#1-#18 sequences around the common sites. Those of D-box#1 and #18 are also shown in Fig. 3b, c. (d) Dual luciferase reporter assays by using all the D-box#1-#18 sequences. The effects of DBP and E4BP4 on transcriptional activities were investigated by luciferase reporters each harboring the indicated D-box or its related sequence. The values of the luciferase activity are shown as ratios of bioluminescence signals from firefly luciferase relative to those from renilla luciferase (internal control). The mean of the signal ratios for each reporter in the absence of DBP and E4BP4 was set to 1. Bars and dots represent means and individual data (n = 3 or 1, empty_E4BP4), respectively. See also Fig. 3d.

a

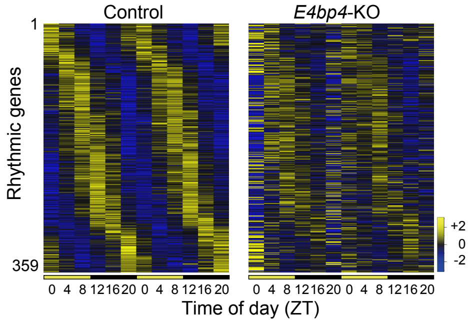

b

Gene Ontology analysis

Term	Count	Percentages	P-value
Metabolic pathways	47	15.6	1.6E-6
Tryptophan metabolism	6	2.0	1.6E-3
Bacterial invasion of epithelial cells	7	2.3	3.0E-3
Fat digestion and absorption	5	1.7	4.9E-3
Biosynthesis of antibiotics	11	3.7	5.8E-3
Bile secretion	6	2.0	9.5E-3

Supplementary Fig. 4 Dysfunction of circadian output in *E4bp4*-KO liver

(a) Heat maps of mRNA levels of 359 genes that showed robust expression rhythms ($p < 0.01$) in the control but lost their rhythmicities ($p \geq 0.05$) in *E4bp4*-KO liver in the RNA-Seq. Genes were ordered by their peak phases in control mice from early day to late night. The FPKM values were normalized so that the mean and the variance were 0 and 3, respectively, for each row of the maps. (b) Gene ontology (GO) analysis of 359 genes that showed robust expression rhythms ($p < 0.01$) in the control but lost their rhythmicities ($p \geq 0.05$) in *E4bp4*-KO liver in the RNA-Seq.

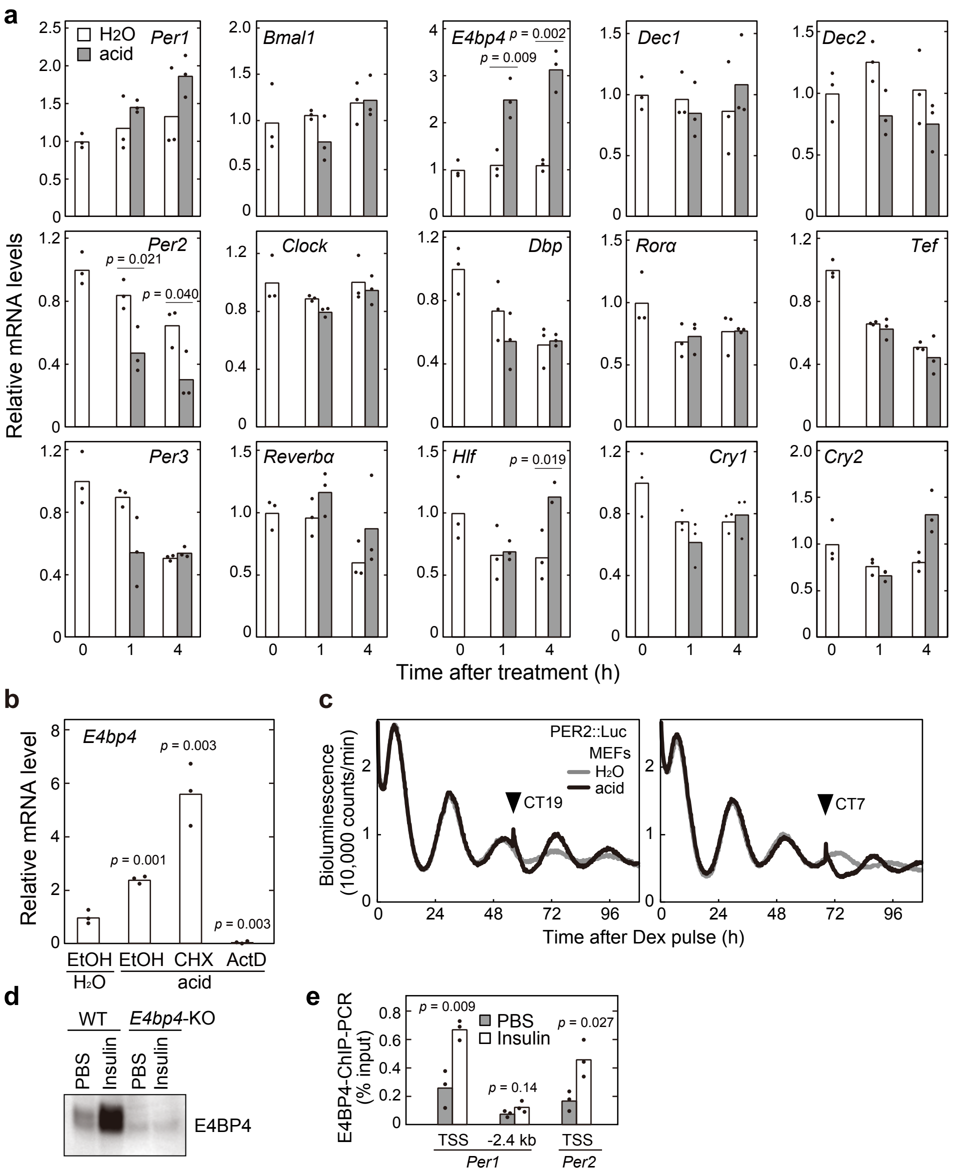

Supplementary Fig. 5 Acute induction of *E4bp4* in response to extracellular stimuli

(a) Changes of mRNA levels of clock genes in response to the acid treatment. MEFs collected 0, 1, 4 hr after the acid treatment were subjected to qRT-PCR analysis. The signal values for each mRNA were normalized to those of *Rps29* mRNA (internal control) and the mean value at time 0 was set to 1. Bars and dots represent means and individual data (n = 3), respectively. The indicated *p* values were calculated by two-sided Student's *t*-test versus H_2O. (b) Effects of cycloheximide and actinomycin D on the acid-induced upregulation of *E4bp4* expression. MEFs were treated with cycloheximide (CHX, final 36 μM), actinomycin D (ActD, final 0.8 μM), or the vehicle (EtOH) 30 min before the acid treatment. The MEFs collected 1 hr after the acid treatment were subjected to qRT-PCR analysis. Bars and dots represent means and individual data (n = 3), respectively. The indicated *p* values were calculated by two-sided Student's *t*-test versus H_2O. (c) Phase-dependent phase shifts of bioluminescence rhythms by the acid treatment in the PER2::LUC MEFs. pHo was changed from 7.0 to 6.6 by adding HCl solution at the different time points indicated by arrowheads. In control experiments, the same volume of water was added (H_2O). (d, e) E4BP4-KO and control mice were fasted for 6 hr starting at ZT 0, and were intraperitoneally injected with insulin (2 units/kg) or PBS at ZT6. Liver nuclear extracts were prepared at ZT 8 (2 hr after the insulin treatment), and were subjected to immunoblot analysis with an anti-E4BP4 antibody (d) and to ChIP-PCR analysis by using an anti-E4BP4 antibody in the *Per1* and *Per2* gene loci (e). TSS refers to transcription start site. Bars and dots represent means and individual data (n = 3), respectively. The indicated *p* values were calculated by two-sidsd Student's *t*-test versus PBS. Full images of the blots are shown in Supplementary Fig. 8.

MOCCS

Sequence	Normalized AUC	Count	MOCCS Rank
CACGTG	18.8	2,591	1
CACGTT	11.6	910	2
CACATG	10.1	2,873	3
CACGCG	9.9	1,607	4
CACGAG	8.1	1,034	5
CATGCG	7.4	738	6
TACGTA	6.9	76	7
CACGGG	6.2	1,388	8

MOCCS2

Sequence	MOCCS2 score	MOCCS2 Rank
CACGTG	102.8	1
CACGTT	37.7	4
CACATG	58.1	2
CACGCG	42.4	3
CACGAG	28.1	5
CATGCG	21.6	7
TACGTA	6.5	-
CACGGG	24.6	6

Supplementary Fig. 6 MOCCS2 analysis of previous CLOCK ChIP-Seq data

The results in our previous MOCCS analysis for CLOCK ChIP-Seq (Yoshitane *et al*., MCB, 2014) were reanalyzed via MOCCS2.

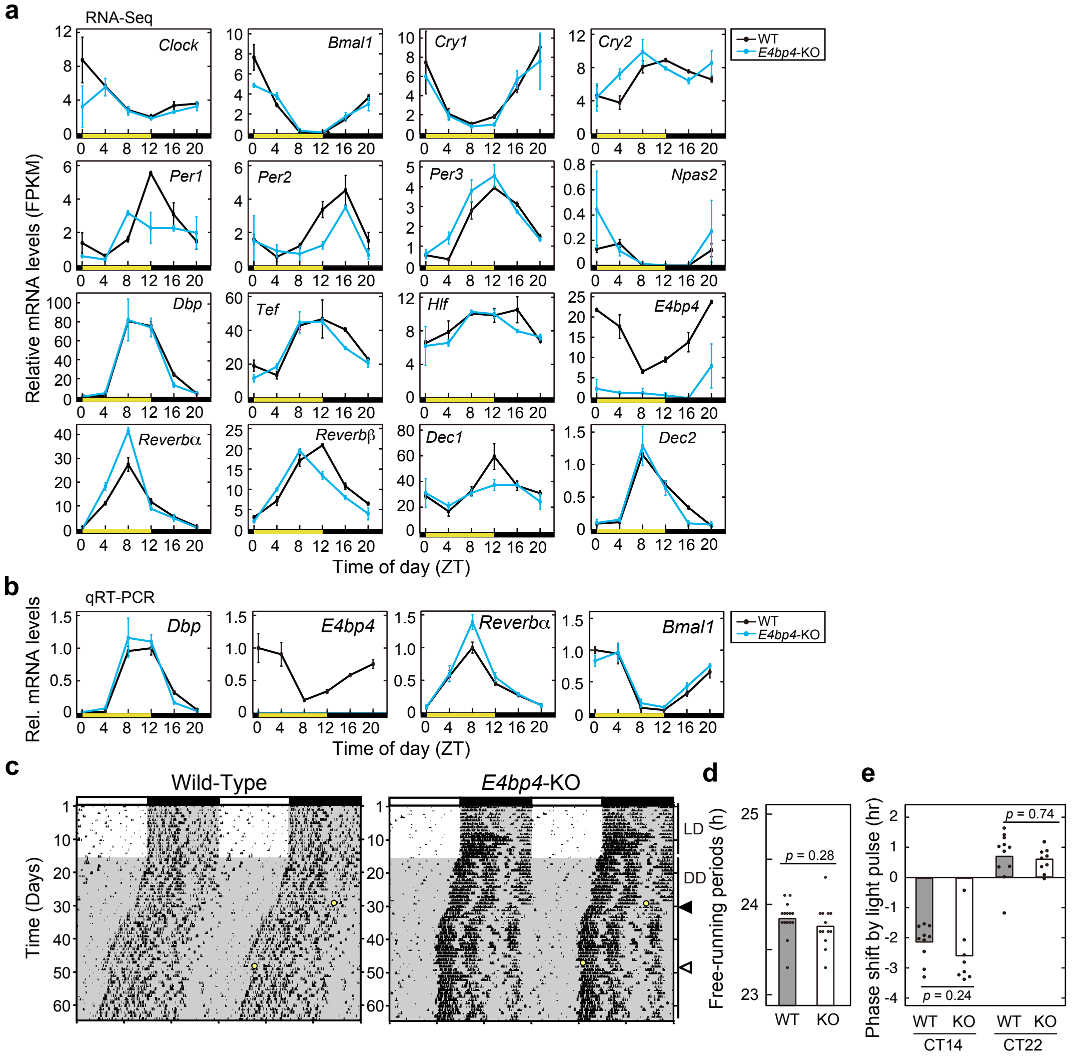

Supplementary Fig. 7 Normal circadian oscillation in *E4bp4*-KO mice

(a, b) Temporal profiles of mRNA levels of indicated core clock genes in livers of *E4bp4*-KO and control mice, determined by RNA-Seq (a) and qRT-PCR analyses (b). Data are means with SD (n = 2) for RNA-Seq and are means with SEM (n = 3) for qRT-PCR. (c) Representative double-plotted locomotor activity records of wild-type (left) and *E4bp4*-KO mice (right). The mice were entrained to 12-h light:12-h dark (LD) cycles and then transferred to constant darkness (DD). A brief light pulse (200 lux, 30 min) was given at CT14 (on the day indicated by a solid arrowhead) and CT22 (an open arrowhead), which caused a phase-delay and a phase-advance, respectively. A gray background indicates the dark period. (d) The periods of the free-running rhythms in WT (black; n=16) and *E4bp4*-KO (white; n=14) mice. Bars and dots represent means and individual data, respectively. The indicated *p* value was calculated by two-sided Student's *t*-test. (e) Magnitudes of the phase shifts caused by the light pulse. By convention, phase delays and advances were shown by negative and positive values, respectively. Bars and dots represent means and individual data (n = 8-11), respectively. The indicated *p* values were calculated by two-sided Student's *t*-test.

Supplementary Fig. 8

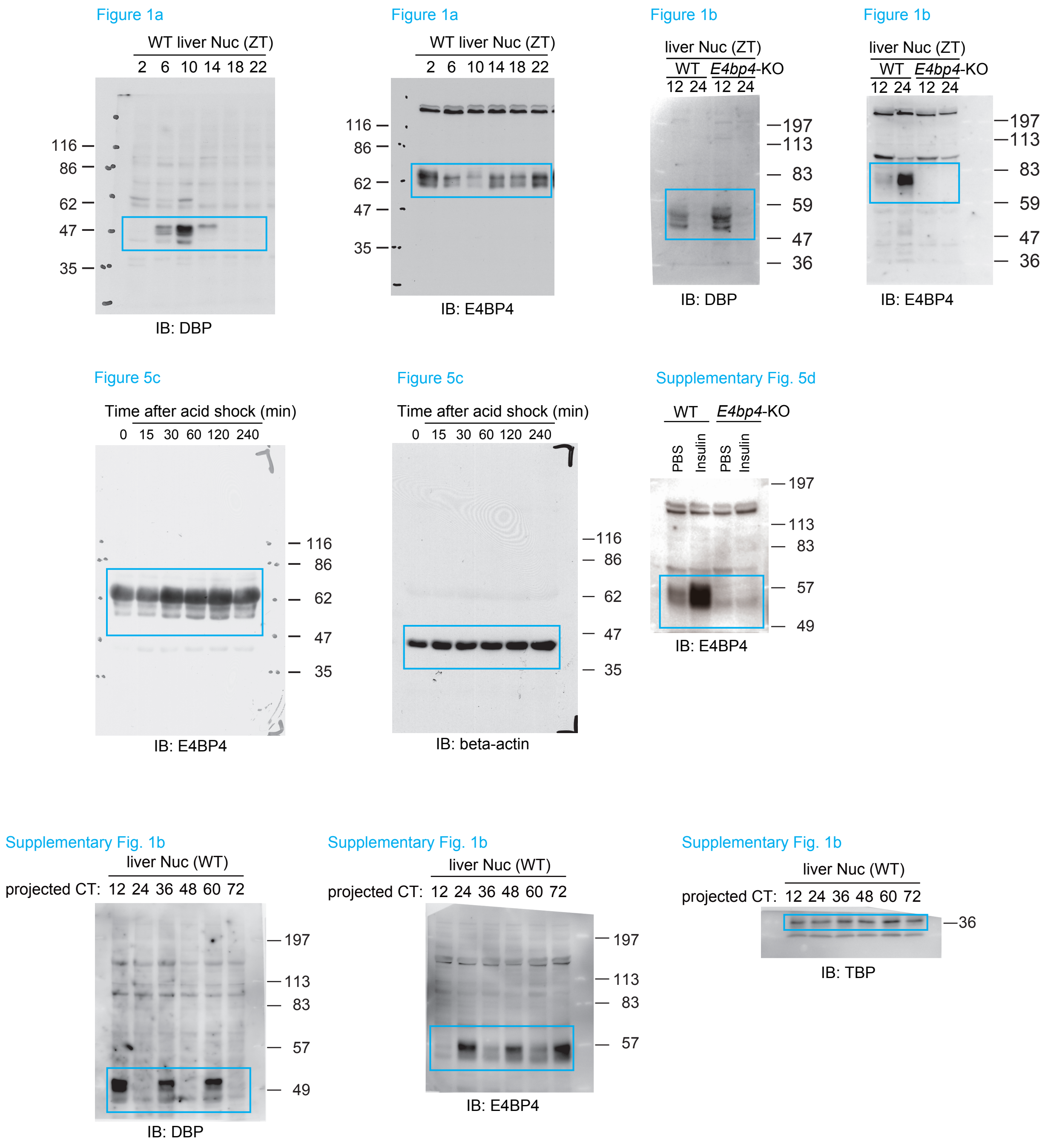

Supplementary Fig. 8 Full images of all the western blots data shown in the manuscript

Supplementary Table 1: List of primers used in the study.

Primer	Sequence
Per1_TSS-Fw	CAGGTAGGGATCCCTGCTGC
Per1_TSS-Rv	ATCAC GTTGG AAGTA GTGGC
Per1_-4.2kb-Fw	CTCTT GTAAT GCCAG AGTC
Per1_-4.2kb-Rv	ACGAC ACTTA CCCAA TACTG
Per1_-2.8kb-Fw	AGAGA AGGAG ACGGA ATTGG
Per1_-2.8kb-Rv	TAATG ATGGC TTGTC CTGC
Per2_TSS-Fw	GCCAT TGGTC GGAGT GCCAC
Per2_TSS-Rv	AGTTC CATGT GCGTC TTATG
Per2_+2.7kb-Fw	ACTTC CTGGT AAGAG CCAAC
Per2_+2.7kb-Rv	TCCTC CCTTA CCCAA CCGTC
Marveld1_TSS-Fw	CTCCT ACATG CCAGG CTCAG
Marveld1_TSS-Rv	AGGTT GAGCT CCCCA CATTG
Wee1_TSS-Fw	TGTTA AATCC GGTGT CCGGG
Wee1_TSS-Rv	GTCCA GGGGG TTGGA ACAAA
Dbp-Fw	AATGA CCTTT GAACC TGATC CCGCT
Dbp-Rv	GCTCC AGTAC TTCTC ATCCT TCTGT
E4bp4-Fw	ATCGG AACAC TGGCA TCAC
E4bp4-Rv	TATCT GACTA CACGC CAGGC
Rev-erba-Fw	AGAAT GTTCT GCTGG CATGT C
Rev-erba-Rv	TTGAG CTTCT CGCTG AAGTC
Bmal1-Fw	TGGTA CCAAC ATGCA ATGC
Bmal1-Rv	AGTGT CCGAG GAAGA TAGCTG
Marveld1-Fw	TGGTG CCACC TCTAA GGTTT CC
Marveld1-Rv	GCAGC GAAGA CTGCC AAACC
Wee1-Fw	TGGCT CTGTT GATGA GCAGA ATG
Wee1-Rv	GCCCA GGCAG AGAAA TAGCG
Rps29-Fw	TGAAG GCAAG ATGGG TCAC
Rps29-Rv	GCACA TGTTC AGCCC GTATT
D-box#1	GATCG TTATG CAACG ATCGT TATGC AACGA TCGTT ATGCA AC
D-box#2	GATCG TTATG TAACG ATCGT TATGT AACGA TCGTT ATGTA AC
D-box#3	GATCG TGATG TAACG ATCGT GATGT AACGA TCGTG ATGTA AC
D-box#4	GATCG TTATG TCACG ATCGT TATGT CACGA TCGTT ATGTC AC
D-box#5	GATCG TTGTG TAACG ATCGT TGTGT AACGA TCGTT GTGTA AC
D-box#6	GATCG TTATA CAACG ATCGT TATAC AACGA TCGTT ATACA AC
D-box#7	GATCG TTAGG TAACG ATCGT TAGGT AACGA TCGTT AGGTA AC
D-box#8	GATCG TGATG CAACG ATCGT GATGC AACGA TCGTG ATGCA AC
D-box#9	GATCG TTATC TAACG ATCGT TATCT AACGA TCGTT ATCTA AC
D-box#10	GATCG TTACG CAACG ATCGT TACGC AACGA TCGTT ACGCA AC
D-box#11	GATCG TGCTG TAACG ATCGT GCTGT AACGA TCGTG CTGTA AC
D-box#12	GATCG TTATG AAACG ATCGT TATGA AACGA TCGTT ATGAA AC
D-box#13	GATCG TTGTG CAACG ATCGT TGTGC AACGA TCGTT GTGCA AC
D-box#14	GATCG TTTGG CAACG ATCGT TTGGC AACGA TCGTT TGGCA AC
D-box#15	GATCG TTATA TAACG ATCGT TATAT AACGA TCGTT ATATA AC
D-box#16	GATCG TTACT CAACG ATCGT TACTC AACGA TCGTT ACTCA AC
D-box#17	GATCG TTATT GCACG ATCGT TATTG CACGA TCGTT ATTGC AC
D-box#18	GATCG TTACG TAACG ATCGT TACGT AACGA TCGTT ACGTA AC
1-missmatched D-box-1 (1.4)	GATCG TAATG CAACG ATCGT AATGC AACGA TCGTA ATGCA AC
1-missmatched D-box-2 (0.5)	GATCG TTCTG CAACG ATCGT TCTGC AACGA TCGTT CTGCA AC
