# Supplementary material for: Functional D-box sequences reset the circadian clock and drive mRNA rhythms
Source: Commun Biol. 2019 Aug 8;2:300. doi: 10.1038/s42003-019-0522-3 (PMC6687812; doi:10.1038/s42003-019-0522-3)
Supplement: Supplementary file 2 — Description of additional supplementary items [file 42003_2019_522_MOESM2_ESM.docx]

**Description of additional supplementary items**

**File Name: Supplementary Data 1**

**Description:** DBP-ChIP-Seq and E4BP4 ChIP-Seq Data

**File Name: Supplementary Data 2**

**Description:** MOCCS2 Data of 1,490 DBP/E4BP4 common sites

**File Name: Supplementary Data 3**

**Description:** RNA-Seq Data in E4bp4-KO and the control livers

**File Name: Supplementary Data 4**

**Description:** MOCCS2 Data of DBP/E4BP4 specific sites

**File Name: Supplementary Data 5**

**Description:** ChIP score of DBP-ChIP-Seq and E4BP4 ChIP-Seq analyses
