# Supplementary material for: Functional D-box sequences reset the circadian clock and drive mRNA rhythms
Source: Commun Biol. 2019 Aug 8;2:300. doi: 10.1038/s42003-019-0522-3 (PMC6687812; doi:10.1038/s42003-019-0522-3)
Supplement: Supplementary file 3 — Reporting Summary [file 42003_2019_522_MOESM3_ESM.pdf]

nature research

Corresponding author(s): Yoshitaka Fukada & Hikari Yoshitane

Last updated by author(s): 2019/06/12

Reporting Summary

Nature Research wishes to improve the reproducibility of the work that we publish. This form provides structure for consistency and transparency in reporting. For further information on Nature Research policies, see Authors & Referees and the Editorial Policy Checklist.

Statistics

For all statistical analyses, confirm that the following items are present in the figure legend, table legend, main text, or Methods section.

n/a	Confirmed	
☐	☒	The exact sample size (n) for each experimental group/condition, given as a discrete number and unit of measurement
☐	☒	A statement on whether measurements were taken from distinct samples or whether the same sample was measured repeatedly
☐	☒	The statistical test(s) used AND whether they are one- or two-sided<br>*Only common tests should be described solely by name; describe more complex techniques in the Methods section.*
☒	☐	A description of all covariates tested
☐	☒	A description of any assumptions or corrections, such as tests of normality and adjustment for multiple comparisons
☐	☒	A full description of the statistical parameters including central tendency (e.g. means) or other basic estimates (e.g. regression coefficient) AND variation (e.g. standard deviation) or associated estimates of uncertainty (e.g. confidence intervals)
☐	☒	For null hypothesis testing, the test statistic (e.g. F, t, r) with confidence intervals, effect sizes, degrees of freedom and P value noted<br>*Give P values as exact values whenever suitable.*
☒	☐	For Bayesian analysis, information on the choice of priors and Markov chain Monte Carlo settings
☒	☐	For hierarchical and complex designs, identification of the appropriate level for tests and full reporting of outcomes
☒	☐	Estimates of effect sizes (e.g. Cohen's d, Pearson's r), indicating how they were calculated

Our web collection on statistics for biologists contains articles on many of the points above.

Software and code

Policy information about availability of computer code

Data collection	All softwares used in this study were described in the manuscript.
Data analysis	All softwares used in this study were described in the manuscript.

For manuscripts utilizing custom algorithms or software that are central to the research but not yet described in published literature, software must be made available to editors/reviewers. We strongly encourage code deposition in a community repository (e.g. GitHub). See the Nature Research guidelines for submitting code & software for further information.

Data

Policy information about availability of data

All manuscripts must include a data availability statement. This statement should provide the following information, where applicable:

- Accession codes, unique identifiers, or web links for publicly available datasets
- A list of figures that have associated raw data
- A description of any restrictions on data availability

Illumina sequencing data for the ChIP-Seq and the RNA-Seq are available in the DDBJ/EBI/NCBI databases under the accession numbers PRJDB7796 and PRJDB7789.

Field-specific reporting

Please select the one below that is the best fit for your research. If you are not sure, read the appropriate sections before making your selection.

☒ Life sciences ☐ Behavioural & social sciences ☐ Ecological, evolutionary & environmental sciences

For a reference copy of the document with all sections, see nature.com/documents/nr-reporting-summary-flat.pdf

Life sciences study design

All studies must disclose on these points even when the disclosure is negative.

Sample size	Sample size was determined as N ≥ 8 for animal experiments and N = 3 for others.
Data exclusions	No data were excluded from the analysis.
Replication	We verified the reproducibility of the experimental finding.
Randomization	Allocation in this study is random.
Blinding	Our behavioral analysis is based on automatic calculation with Clocklab, and therefore it does not require blinding.

Reporting for specific materials, systems and methods

We require information from authors about some types of materials, experimental systems and methods used in many studies. Here, indicate whether each material, system or method listed is relevant to your study. If you are not sure if a list item applies to your research, read the appropriate section before selecting a response.

Materials & experimental systems

n/a	Involved in the study	
☐	☒	Antibodies
☐	☒	Eukaryotic cell lines
☒	☐	Palaeontology
☐	☒	Animals and other organisms
☒	☐	Human research participants
☒	☐	Clinical data

Methods

n/a	Involved in the study	
☐	☒	ChIP-seq
☒	☐	Flow cytometry
☒	☐	MRI-based neuroimaging

Antibodies

Antibodies used	Information of all antibodies was described in the manuscript.
Validation	We validated all of the antibodies for their specificities.

Eukaryotic cell lines

Policy information about cell lines

Cell line source(s)	HEK293T/17 used in this study is from ATCC cell bank.
Authentication	Yoshitaka Fukada is authentificated the license of the cell line.
Mycoplasma contamination	We confirmed that the cell line was negative for mycoplasma.
Commonly misidentified lines (See ICLAC register)	n/a

Animals and other organisms

Policy information about studies involving animals; ARRIVE guidelines recommended for reporting animal research

Laboratory animals	Information for animals used in this study was described in the manuscript.
Wild animals	Information for animals used in this study was described in the manuscript.
Field-collected samples	n/a
Ethics oversight	The animal experiments were approved by the animal ethics committee of the University of Tokyo.

Note that full information on the approval of the study protocol must also be provided in the manuscript.

ChIP-seq

Data deposition

☒ Confirm that both raw and final processed data have been deposited in a public database such as GEO.

☒ Confirm that you have deposited or provided access to graph files (e.g. BED files) for the called peaks.

Data access links<br>*May remain private before publication.*	n/a
Files in database submission	Illumina sequencing data for the ChIP-Seq and the RNA-Seq are available in the DDBJ/EBI/NCBI databases under the accession numbers PRJDB7796 and PRJDB7789.
Genome browser session<br>(e.g. UCSC)	n/a

Methodology

Replicates	Biological replicates from two mice.
Sequencing depth	See in Sup Table.
Antibodies	See in the Method section.
Peak calling parameters	See in the Method section.
Data quality	In peak calling analysis, model fold = [5, 50] and qvalue cut off = 1.00e-02. We also confirmed the quality and the enrichment of the samples by ChIP-PCR as main figures.
Software	See in the Method section.
